# Advancing Systemic Risk Assessment for Complex, Interdependent Systems: A Research Agenda

**DOI:** 10.1111/risa.70243

**Published:** 2026-04-14

**Authors:** Tom Logan, B. Rachunok, K. Hardaway, D. Bristow, A. Reilly, D. Johnson, J. Johansson, G. Cremen, J. Boakye, P. Schweizer

**Affiliations:** ^1^ Department of Civil and Environmental Engineering University of Canterbury Christchurch New Zealand; ^2^ Edwards P. Fitts Department of Industrial & Systems Engineering North Carolina State University Raleigh North Carolina USA; ^3^ Department of Civil Engineering University of Victoria Victoria Canada; ^4^ Department of Civil and Environmental Engineering University of Maryland College Park Maryland USA; ^5^ Department of Industrial Engineering and Political Science Purdue University West Lafayette Indiana USA; ^6^ Division of Risk Management and Societal Safety Lund University Lund Sweden; ^7^ Department of Civil Environmental and Geomatic Engineering University College London London UK; ^8^ Department of Civil and Environmental Engineering University of Massachusetts Amherst Amherst Massachusetts USA; ^9^ RIFS Research Institute of Sustainability Potsdam Germany

**Keywords:** cascading effects, complex systems, interdependencies, risk assessment methods, systemic risk, uncertainty

## Abstract

Engineering risk assessment has traditionally focused on direct impacts to individual assets or systems. However, as society's most notable risks increasingly stem from complex, interdependent systems, conventional methods fail to capture the cascading consequences and deepening uncertainty. Addressing this gap requires developing or extending assessment methods. To guide such development and align research efforts, this paper introduces a taxonomy of risk assessment methods. The taxonomy classifies methods by their capability to assess different types of consequences and systemic behaviors. Tier 1 methods can assess direct, localized impacts; Tier 2 methods also capture cascading consequences in interconnected systems; Tier 3 methods also address emergent, transformative, and uncertain dynamics. We identify key system and knowledge characteristics that challenge existing methods and outline research priorities necessary to advance systemic risk assessment: Analyzing consequences, structuring uncertainty, evaluating trade‐offs, strengthening causal inference, ensuring defensibility, and communicating results. This agenda aims to guide future research towards risk assessment methods suitable for the systemic risk challenges society increasingly faces.

## 1 Introduction

Engineering risk assessment has traditionally focused on isolated technical elements and direct system impacts, using quantitative methods to evaluate system reliability and failure probabilities. These methods have served well in bounded contexts, but today's most urgent and consequential risks often originate from outside of narrow system boundaries. Although infrastructure systems have long been intertwined with social, environmental, and economic systems, only recently has there been growing recognition that risk exists not just in individual elements but in complex interactions, both within and across system boundaries. Feedback loops, cascading disruptions, contested values (i.e., disagreement over what outcomes matter), and unintended consequences expose the limits of conventional approaches to risk assessment (Arvidsson et al. 2021; Haimes 2017; Nocera et al. 2025; Sillmann et al. 2022; Simpson et al. 2021; Svegrup et al. 2019).

These limits are further amplified by wicked challenges such as climate change, globalization, urbanization, and digitalization. These changes are introducing uncertainties and system‐wide vulnerabilities that challenge the assumptions underlying conventional risk assessment. For example, most existing methods are designed to evaluate discrete events or component failures within a defined system. But today's risks propagate across domains and produce cascading or emergent consequences that these methods were not built to capture (Cremen et al. 2022b). Simplifying these systems or imposing narrow boundaries for the sake of tractable modeling can misrepresent risk, obscuring the pathways through which failures spread and compound within and across systems. Worse, interventions based on reductionist approaches may lead to maladaptive outcomes by failing to account for system feedbacks or behavioral responses (S. E. Anderson et al. 2018; Barnett and O'Neill 2010; Forrester 1971a; Rittel and Webber 1973).

**FIGURE 1:**
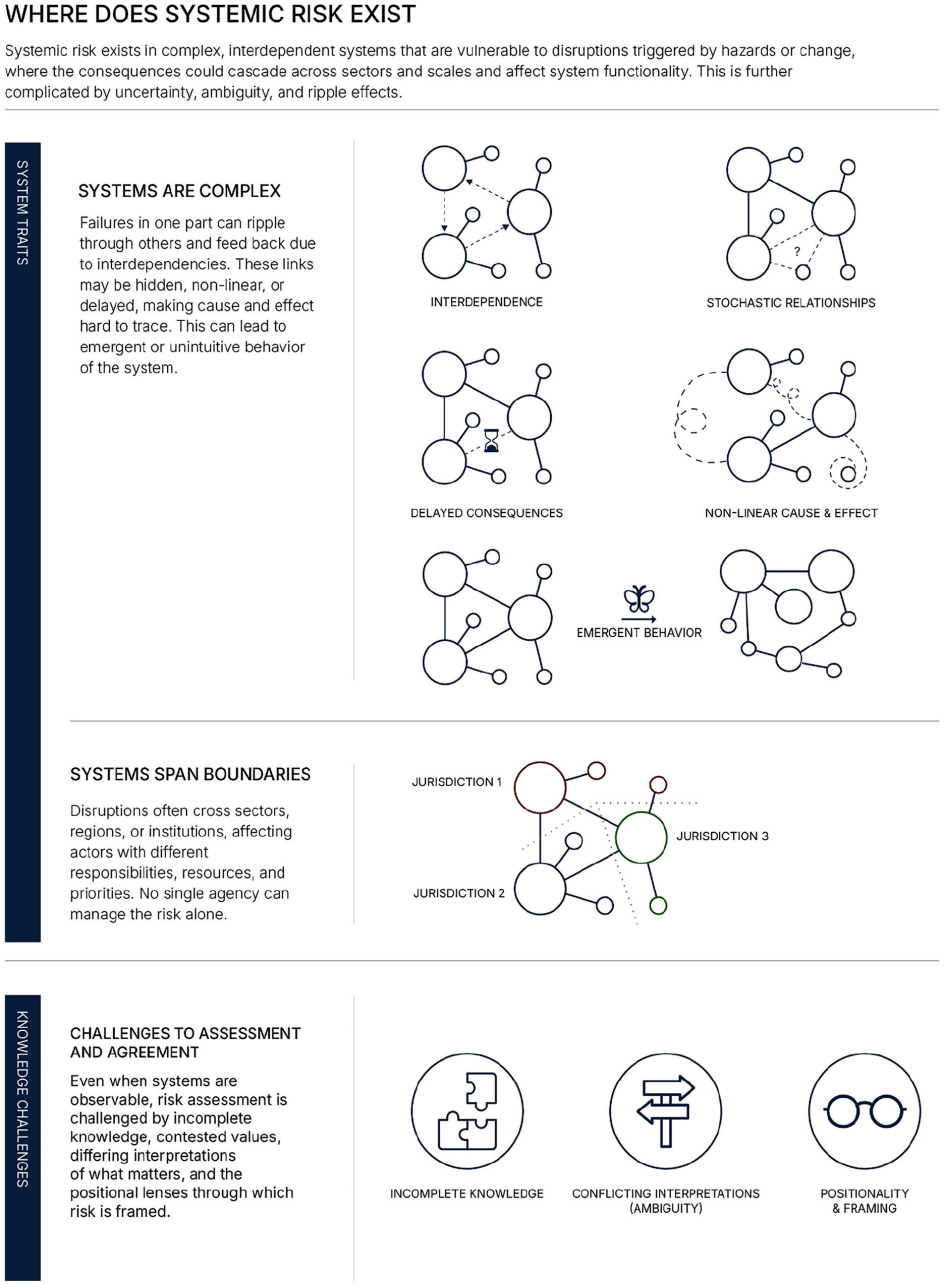
**Conditions of systemic risk**. Systemic risk exists when system traits and knowledge challenges interact in complex, interdependent systems. Traits such as complexity and multidimensional consequences shape how disruptions could propagate and transform. Knowledge challenges, including ambiguity, incomplete knowledge, and the unintended effects of action, further complicate assessment and response. Together, these conditions create challenges for traditional risk assessment approaches.

We argue that engineering risk assessment must evolve to explicitly account for the complexity of modern systems. In doing so, we adopt a conceptually broad understanding of risk, in which risk refers to uncertainty about and severity of the consequences of an activity with respect to something that humans value (Aven and Renn 2009; SRA 2022). More specific formulations (such as scenario‐based, probabilistic, or expected‐loss representations) are understood as particular ways of characterizing uncertainty and consequence, rather than as fundamentally different definitions of risk. Within this framing, systemic risk refers to a subset of risk that exists in complex, interdependent engineered, social, and political systems, where uncertainty about and severity of consequences are shaped by cascading and potentially amplifying pathways across sectors and scales (Figure 1; Table 1). It is marked by uncertainty and ambiguity beyond the point of origin (Johansson et al. 2015; Liu and Renn 2025; Pescaroli and Alexander 2018; Renn et al. 2022; Schweizer and Renn 2019; Sillmann et al. 2022).

**TABLE 1:** **System properties and knowledge challenges that matter for understanding systemic risk**. Each property influences how disruptions unfold and what makes risk hard to trace, assess, or manage.

	Trait/Challenge	Description
**System traits**	**Temporal dynamics**	System change over time, with path dependencies, delayed effects, and changing baselines. Infrastructure and regulations suited for past conditions may fail under future extremes, e.g., (Haimes 2017; Hardaway and Flage 2025; Logan et al. 2021; Meyer and Johnson 2019; Nasr et al. 2023; Otárola et al. 2024).
	**Spatial variation**	Consequences differ across locations depending on exposure, capacity, and vulnerability. Spatial averaging often obscures place‐specific dynamics, e.g., (Argyris et al. 2019; Chen et al. 2022).
	**Multidimensional consequences**	Impacts can be financial, physical, social, and intangible. These are hard to compare and raise normative questions about which outcomes matter most, e.g., (Boakye et al., 2022; Fekete, 2011; McCloskey et al., 2023; Peacock et al., 2012)
	**Transboundary**	Impacts cross jurisdictional boundaries, complicating accountability, planning, and coordination, e.g., (Lawrence, Blackett, et al. 2020; Renn et al. 2022).
	**Multiple actors**	Diverse stakeholders with differing values and capacities may shift or exacerbate risk to others impacted by the same system through indirect or conflicting actions (D. R. Johnson et al. 2023; Schweizer 2021).
	**Causal complexity**	Cause–effect relationships may be delayed, non‐linear, and stochastic. Interdependence, feedback loops, and emergent behavior exacerbate risk and complicate assessment (Forrester 1971b; Haimes 2017; Liu and Renn 2025; Ramaswami et al. 2018; Sillmann et al. 2022).
**Knowledge challenges**	**Uncertainty**	Risk is shaped by incomplete knowledge (epistemic) and inherent variability (aleatory). Surprises can arise when models omit key dynamics and rely on assumptions (Aven 2013a, 2013b; Cox 2012; Hardaway and Flage 2025; Marchau et al. 2019; Paté‐Cornell 1996; Walker et al. 2003).
	**Ambiguity**	Different actors may have different, legitimate perspectives that cannot be resolved by more data or technical analysis alone, e.g., (Cedergren et al. 2018; Florin and Bürkler 2017; Renn and Schweizer 2009).
	**Effect of actions**	Interventions can shift or exacerbate risk, sometimes in counterintuitive ways. Fixes in one part of the system may create new vulnerabilities elsewhere, e.g., (S. E. Anderson et al. 2018; Forrester 1971a; Logan et al. 2018; Nocera et al. 2025; Rachunok and Nateghi 2021; Rittel and Webber 1973).
	**Positionality and framing**	Risk assessments are shaped by the values, assumptions, and institutional perspectives of those conducting them. These choices influence what is modeled, whose knowledge is included, and how risk is interpreted (Fraser 2001; Schweizer 2021; Williams et al. 2022).

These dynamics are especially salient in the context of climate adaptation. As an illustrative example, consider an extreme event that disrupts the power grid. Power outages, in turn, affect water treatment, healthcare, transport, and communications, each disruption compounding the others, delaying recovery, amplifying consequences, and shifting the burden onto vulnerable populations (M. J. Anderson et al. 2025; Brunner et al. 2024; Centeno et al. 2015; Rinaldi et al. 2001; Svegrup et al. 2019). In such cases, risk is not the sum of isolated failures, but the result of complex pathways through which disruptions cascade and compound. Many engineered elements and systems were planned and assessed for risk independently but failed to account for the emergent vulnerabilities that arise when these operate within an interdependent systems context. Risk assessments that overlook cross‐sectoral and scale dependencies, such as the centrality of electricity to healthcare, mobility, and communication, risk missing critical vulnerabilities (M. J. Anderson et al. 2025; Essus and Rachunok 2024; Svegrup et al. 2019). Going unsaid in this example is the fact that climate change also introduces new risks, as, for example, extreme heat events not only pose challenges to power grid stability but present an opportunity for an adversary to strike industrial control systems, given the greater potential for disastrous consequences in an already destabilized power system (D. Johnson et al. under review; Sobczak and Behr 2016).

Importantly, the models and metrics we use to assess risk do more than describe the system, they can shape how the problem is framed, what outcomes are made visible, and which decisions are considered legitimate (Slovic 1999). Risk assessments can unintentionally exclude groups or prioritize values aligned with the modeler's assumptions, reinforcing inequities in who is protected or burdened. Choices about what is modeled, what is measured, and whose perspectives are represented directly influence the results, and, in turn, the decisions they inform (Fraser 2001; Tan et al. 2024; Williams et al. 2022).

So how can we advance engineering risk assessment to address systemic risk, where risk exists in complex, interdependent systems and is shaped by feedback loops and diverse perspectives? This paper introduces a taxonomy of assessment methods and a research agenda to guide their development. This taxonomy classifies methods by their capability to assess different orders of systemic consequences and uncertainty, identifies limitations of current approaches in this context, and outlines research priorities for the field.

Our aim is to structure a body of knowledge for systemic risk assessment, providing a foundation that researchers and practitioners can build on. While our emphasis is on assessment methods, we acknowledge the broader literature on the governance, perception, and communication of systemic risk, which offers complementary perspectives beyond this paper's scope (e.g., Schweizer 2021; Florin and Bürkler 2017; Renn et al. 2022; Sillmann et al. 2022; Rydén Sonesson et al. (2021).

## 2 Complexity in Risk: What Assessment Must Now Address

To advance systemic risk assessment, we need a shared language that describes how risk propagates through complex, interdependent systems and can guide the development of assessment methods according to the complexity of consequences they can evaluate.

The foundational frameworks for risk assessment require extension to meet this challenge. The foundational framing risk by Kaplan and Garrick (1981), extended by Haimes (2009), provides structure but assumes bounded systems with traceable consequences and quantifiable uncertainties. Analysis of systemic risk requires frameworks that address cascading dynamics, intervention effects, and multiple perspectives. We therefore propose seven revised and expanded questions to guide risk assessment methods:

1. What can go wrong?
  Failures not only arise in isolation but from cascades across interdependent systems.
2. What are the consequences?
  Consequences extend beyond direct impacts to include higher‐order effects that cascade across systems, affecting multiple actors and jurisdictions.
3. What is the likelihood?
  In contexts of deep uncertainty, traditional probabilistic methods may fail to capture what is unknown or in flux. Both aleatory and epistemic uncertainties must be represented in transparent, decision‐relevant ways.
4. Over what timeframe?
  System dynamics unfold across multiple horizons, from immediate to long‐term. Risk assessments must account for temporal dynamics, including time lags and evolving vulnerabilities.
5. How might actions change the system?
  Interventions can trigger unintended consequences or lock‐in effects. Understanding intervention effects requires causal reasoning and exploration of adaptive pathways.
6. What assumptions shape our model of the system?
  All models are value‐laden. Defensible assessments must clarify framing, positionality, and underlying assumptions: What is included, what is left out, and why.
7. Importance for whom?
  Risk is not objective or universal. It is experienced differently across actors and shaped by power and place. Identifying whose risks are modeled is essential to recognitional justice and legitimacy.

**FIGURE 2:**
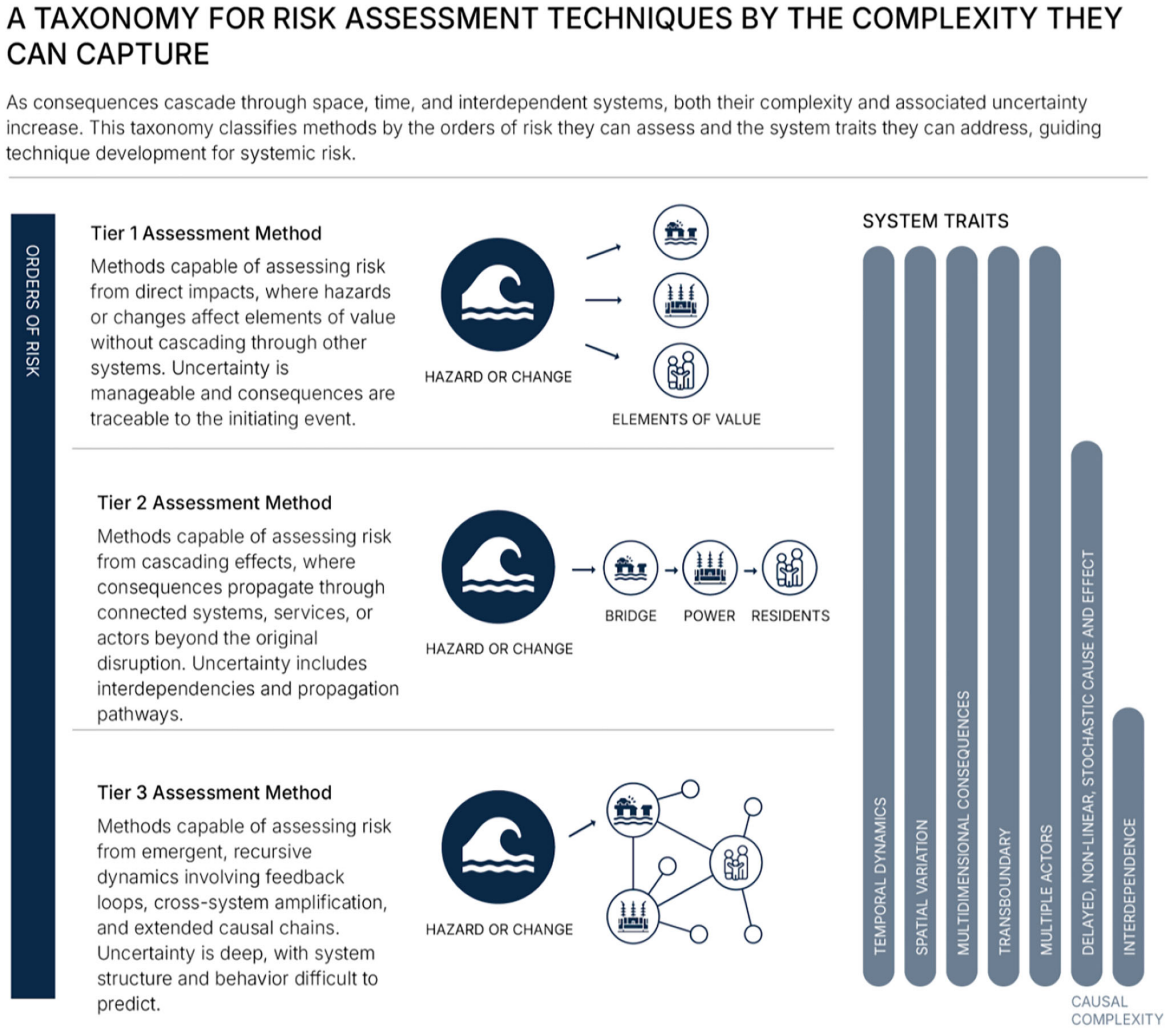
A taxonomy of risk assessment methods by capability. The taxonomy classifies methods by their capacity to assess consequences and uncertainty as they increase in complexity. Tier 1 methods assess direct impacts with often manageable uncertainty. Tier 2 methods capture cascading effects and interdependencies with increasing levels of uncertainty. Tier 3 methods address emergent dynamics and deep uncertainty.

These questions guide our taxonomy of risk assessment methods. We introduce “Risk Assessment Tiers,” a taxonomy that classifies methods by their capability to assess higher‐order consequences and represent associated uncertainties. Each tier corresponds to methods suited to different orders of systemic consequence and engages differently with the six system traits and four knowledge challenges outlined earlier. While any approach's performance depends on how system boundaries are defined, the tiers reflect what different methods are designed to handle. By linking methodological capability to consequence complexity and uncertainty, this taxonomy guides the development and selection of approaches for systemic risk assessment (Figure 2).

These capability distinctions reflect a pattern in how risk manifests. As consequences cascade, their complexity and associated uncertainty increase. Risk propagation can be initiated by acute hazards or threats (such as earthquakes or cyberattacks) or by chronic changes that gradually alter vulnerabilities, such as sea‐level rise or evolving infrastructure dependencies. In both cases, effects rarely stop at the point of direct impact. First‐order consequences often trigger second‐order consequences in connected systems, which can generate third‐ and higher‐order consequences as disruptions cascade further (Rinaldi et al. 2001). As consequences propagate to higher orders feedback loops and emergent behavior appear, while uncertainty shifts from well‐characterized variability to deep uncertainty about system structure and response. These dynamics challenge conventional assessment methods designed for bounded, traceable risks. The taxonomy hence classifies methods by their capability to trace cascading consequences and represent the deepening uncertainty. Higher‐order consequences are also those most likely to cross jurisdictional boundaries, complicating both risk assessment and management where regulations may restrict what can be considered.

The following sections describe each tier, illustrating what each type of method can assess and where capabilities fall short. This clarifies both which existing methods are fit for specific contexts and where new development is needed.

### 2.1 Tier 1 Assessment Methods

Tier 1 methods are used to assess direct risks, where a disruptive event immediately affects one or more elements of value, including physical assets, natural environments, cultural sites, social systems, or essential services. These consequences occur without intermediary propagation, and causal relationships are typically straightforward and traceable.

While direct risks may still vary across space, time, and context, they do not involve cascading effects, meaning they cannot address the causal complexity challenge. From an assessment perspective, direct and first‐order consequences are analytically equivalent: They require methods capable of capturing immediate impacts, often with attention to spatial variation, value dimensions, or context‐specific thresholds. Tier 1 methods are characterized by their focus on localized cause–effect relationships within a single system or domain.

Example: A tsunami inundates a coastal region, directly damaging several bridges along a highway. Though the assets span multiple jurisdictions and serve different user groups, the damage is attributable solely to the hazard event, without intermediate disruption through other systems. Tier 1 methods for this scenario include exposure‐damage assessments and direct loss estimation based on replacement costs.

Tier 1 methods are defined by their capability to address these characteristics of direct risk, and are limited to localized, non‐cascading consequences.

*Specifically, Tier 1 Methods can address the following system traits:*

- Spatial variation: Consequences differ depending on asset location, design, governance and event severity. While Tier 1 methods must account for geographic differences in exposure and vulnerability, consequences remain localized to directly affected elements.
- Temporal Dynamics: Risk changes over time due to the asset condition (e.g., age or prior stress) or shifts in event frequency and severity.
- Multidimensional consequences: Impacts may include physical, financial, social, or environmental dimensions, each valued differently by different actors.
- Transboundary: A single event may simultaneously impact multiple sectors, owners, or jurisdictions.
- Multiple Actors: Risk involves diverse stakeholders who may own, use, or be affected by the same elements or services.

*Tier 1 Methods can address the following knowledge challenges:*

- Uncertainty: Element or event characteristics may be incomplete or variable.
- Ambiguity: Stakeholders may disagree on which outcomes matter most or how to interpret severity.
- Effect of actions: While more predictable than in higher tiers, interventions still shape consequences and may introduce path dependencies.
- Positionality and framing: Model assumptions reflect whose outcomes are measured and which indicators define severity or success.

### 2.2 Tier 2 Assessment Methods

Tier 2 methods are used to assess second‐order and cascading risks associated with direct impacts disrupting connected systems or sectors. These methods capture consequences that propagate through linear or branching pathways, even across multiple steps, while remaining causally traceable to the initiating event. We define Tier 2 methods as those that cannot model recursive feedbacks, compounding dynamics, or emergent behaviors. They are suited to contexts where risk accumulates through traceable, additive or sequential pathways, but not where impacts amplify nonlinearly or trigger changes to system structure.

Example: Damage to coastal bridges from a tsunami disrupts inland access to healthcare facilities. Although the hospitals are not directly affected, their functionality is compromised due to road outages, delaying emergency response and critical care. A Tier 2 method could assess this risk, as it results from a discernible chain of indirect effects, without involving feedback or systemic escalation. Tier 2 methods for assessing such cascading effects include dependent network models such as infrastructure service disruption analysis, and indirect casualty assessment (e.g. lives lost due to delayed emergency response).

Tier 2 methods are defined by their capability to address the same system traits and knowledge challenges introduced in Tier 1, but in more complex and distributed forms, including cascading consequences across interconnected systems. Additional traits and challenges addressed by Tier 2 methods are outlined below.

*In addition to those in Tier 1, Tier 2 methods address the following system traits:*

- Causal complexity (newly introduced at Tier 2): Consequences are quantified from connections between systems and elements. Unlike Tier 1's direct cause–effect relationships, Tier 2 methods must trace how disruptions propagate through multiple systems in sequence, requiring data on direct dependencies and propagation mechanisms.
- Spatial variation: Consequences may manifest in areas not directly affected by the initiating event.
- Temporal dynamics: Effects may be delayed, unfolding over hours, days, or even longer after the initial event.
- Multidimensional consequences: Impacts increasingly include intangible or systemic effects across systems (e.g., service disruption, behavioral shifts).
- Transboundary: Risk propagates across sectoral or jurisdictional boundaries, complicating coordination among disparate institutions.
- Multiple actors: Affected parties may be disconnected from the initial impact, introducing challenges for coordination, accountability, and shared understanding.

*In addition to those in Tier 1, Tier 2 methods address the following knowledge challenges:*

- Uncertainty: Includes uncertainty not just about exposure or event and associated vulnerability, but about how systems interact and respond under stress and the subsequent consequences.
- Ambiguity: Greater separation from the source event increases disagreement over which impacts matter, who is responsible, and how to evaluate or prioritize risk.
- Effect of actions: Interventions may trigger unintended outcomes due to indirect pathways or lagged effects.
- Positionality and framing: Framing shapes what systems are linked and which effects are considered, influencing who is seen as affected and how responsibility is attributed.

### 2.3 Tier 3 Assessment Methods

Tier 3 methods are used to assess higher‐order risks, where extended chains of impact involve feedback loops, cross‐sector amplification, and emergent behavior. These risks cannot be adequately captured through linear propagation alone. Instead, they require methods that can represent recursive dynamics, nonlinear escalation, and/or structural shifts in system function, conditions under which cause and effect may become intertwined and outcomes evolve endogenously. Because these methods address feedback loops, they may also be able to more accurately capture additional impacts that lower‐tier methods would not capture.

Example: A tsunami damages key bridges, disrupting access to inland hospitals. One bridge also carries power lines, cutting electricity to construction supply depots and equipment yards. These facilities are critical to enabling bridge repair, so the power outage delays restoration, prolonging hospital inaccessibility and compounding the original disruption. The cascading impact loops back into the system, creating a feedback cycle that escalates the consequences and complicates recovery. Tier 3 methods capable of capturing these feedback dynamics include system dynamics models that incorporate positive feedback loops associated with power outages, agent‐based models that capture emergent behaviors of construction crews adapting to these outages, and matrix or network‐based approaches that also capture the feedback or adaptation processes.

Tier 3 methods are defined by their capability to address the system traits and knowledge challenges present in Tiers 1 and 2 as they evolve in form or intensity, including recursive, adaptive, and emergent dynamics. Causal complexity becomes mutual rather than unidirectional, and relationships between system components may shift dynamically. Tier 3 methods are distinguished by their ability to capture these processes, where the system itself is transformed in response to disturbance.

*In addition to those in Tiers 1 and 2, Tier 3 methods address the following system traits:*

- Causal complexity (interdependence arises): Systems are mutually dependent, making causal chains recursive, opaque, and difficult to isolate. Unlike Tier 2's traceable propagation pathways, Tier 3 dynamics involve feedback loops where consequences feedback to reshape the original system, creating cyclical or amplifying effects that cannot be captured through linear interactions.
- Temporal dynamics: Recursive impacts can generate long‐term or cyclical consequences, where earlier disruptions re‐emerge or worsen over time.
- Multidimensional consequences: Impacts are shaped by emergent behaviors and feedback loops, complicating attribution and prioritization.

*In addition to those in Tiers 1 and 2, Tier 3 methods address the following knowledge challenges:*

- Uncertainty: Risk becomes highly sensitive to interacting variables, lagged effects, and unknown tipping points. Small changes in inputs can lead to divergent outcomes.
- Ambiguity: Nonlinear dynamics and systemic coupling lead to conflicting interpretations about causality, responsibility, and acceptable trade‐offs.
- Effect of actions: Interventions may backfire, producing destabilizing, counterintuitive, or maladaptive outcomes. Fixes in one domain can exacerbate vulnerabilities elsewhere or entrench systemic fragility.
- Positionality and framing: At this tier, framing and positionality define the system itself, what feedbacks are modeled, which futures are considered, and whose resilience is prioritized.

It is important to note that the taxonomy classifies methods by their capability to assess specific risk dynamics, not by method type or label. A given modeling approach, such as network analysis or agent‐based modeling, may be applied at different tiers depending on what it represents. A network model, for instance, that traces direct impacts is Tier 1; one that captures linear cascading disruptions is Tier 2; one that includes feedback loops and adaptive responses is Tier 3. We also emphasize that this is a hierarchy of risk assessment methods, not a hierarchy of risks. We are not being prescriptive in characterizing risks or systems as being Tier 1, 2 or 3; as in the tsunami example, the appropriate choice of risk assessment method is guided by the choice of impact(s) to be analyzed and an expert judgment about which of the system traits and knowledge challenges are relevant for the analysis.

## 3 Priorities for Advancing Risk Assessment in Complex Systems

Most current practically applied assessment approaches rarely extend beyond Tier 1 or basic Tier 2 capabilities. A growing mismatch exists between the complexity of risk and the capability of prevailing assessment methods. This section identifies priority areas for advancing systemic risk assessment. Rather than reviewing existing methods, we focus on the analytical capabilities needed to evaluate cascading and emergent consequences and their associated uncertainties. These priorities are derived from the limitations identified through the Risk Assessment Tiers and are linked to the questions posed in Section 2. Each priority highlights where new method development or empirical research is needed to capture cascading and emergent consequences, represent deep uncertainty, and support defensible decisions in complex contexts.

### 3.1 From Isolated Impacts to Cascading and Emergent Consequences

Consequences in complex systems often extend far beyond the initial point of impact. A disruptive event may trigger follow‐on consequences that ripple through interdependent systems and evolve over time. These cascading and emergent outcomes are difficult to anticipate and quantify, yet they frequently determine the overall burden of risk. Many assessment methods remain focused on direct, first‐order impacts, with limited ability to capture how consequences unfold across space, time, and domains.

This priority engages with the guiding question “What are the consequences?” in contexts where impacts propagate through networks or emerge through feedback loops. Tier 2 and Tier 3 methods are those that can assess these dynamics, moving beyond immediate damage to evaluate how consequences escalate, or transform system behavior over time.

Example: After a severe rainfall event in a rural region, a single bridge on a key arterial road was damaged. While no homes were inundated and utilities remained operational, the disruption severed access to several communities. Emergency services were delayed, supply chains rerouted, and residents experienced extended isolation. The measurable “damage” was minimal. Yet the true burden, emotional distress, loss of mobility, and institutional strain far exceeded what conventional assessments capture.

Empirical studies demonstrate how traditional risk assessment underrepresents risk in complex systems, particularly when consequences propagate through systemic dependencies and nonlinear interactions (M. J. Anderson et al. 2024, 2025; Best et al. 2023; Brunner et al. 2024; Dawson et al. 2018; Hochrainer‐Stigler et al. 2023; Johansson et al. 2015; Logan et al. 2023; Ouyang 2014; Ouyang and Wang 2015; Svegrup et al. 2019; Thompson et al. 2024).

Emergent consequences add another layer of complexity. These outcomes result not from a linear cause and effect but from interacting components and latent dependencies: Dynamics that are not captured with Tier 1 or 2 methods and that also contain significant uncertainty (Reilly et al. 2021). For example, levee‐reinforcement models may suggest reduced flood risk but fail to capture induced development in flood‐prone areas, increasing long‐term exposure (Barnett and O'Neill 2010; Logan et al. 2018). Frameworks such as socio‐environmental‐technical systems (SETS) offer a starting point for representing feedbacks among infrastructure, human behavior, and environmental change, but operationalizing these dynamics in defensible assessments remains a challenge.

Temporal and spatial variation further complicate consequence analysis. The same disruption may have minimal impact in one setting but catastrophic effects in another, depending on system configuration, redundancy, buffers, time of year, and social vulnerability. Urban systems behave as complex, adaptive networks where adaptation in one area can displace risk or generate new vulnerabilities elsewhere (Bai et al. 2018; Hummel et al. 2021; Ouyang and Dueñas‐Osorio 2012). Conventional Tier 1 methods often collapse these nuances, masking vulnerable elements and distorting priorities (Argyris et al. 2019; Lawrence, Blackett, et al. 2020; Logan et al. 2021).

To address these challenges, risk assessment must trace how consequences propagate and evolve, rather than simply estimating direct impacts. Promising approaches include stakeholder‐informed system mapping (engaging local actors to explore dependencies and impact pathways), dynamic simulation (capturing time‐dependent feedbacks and evolving conditions), and network analysis (identifying nodes and links critical to cascading failures) (Cradock‐Henry et al. 2020; D. R. Johnson 2021; Ramaswami et al. 2018; Svegrup et al. 2019).

Understanding how consequences evolve, who they affect, and how they amplify is foundational for systemic risk assessment. Without these insights, assessments may underestimate the burden of complex events, misalign investments, and overlook systemic vulnerabilities.

### 3.2 From Single Objectives to Multi‐Criteria and Participatory Evaluation

Risk does not affect everyone equally, and its evaluation cannot be reduced to a single metric. In interdependent systems, consequences span economic, social, cultural, and institutional domains, each valued differently by different actors. Yet many assessment methods still prioritize technical performance or cost‐effectiveness alone, overlooking competing priorities such as cultural identity, mobility, equity, or wellbeing (Boholm 2015; Boholm and Corvellec 2011; Pelling et al. 2022). As a result, key trade‐offs remain unexamined, and assessments risk privileging certain worldviews while marginalizing others.

This priority addresses the question “Importance for whom?” Methods must engage with plural values and perspectives to address systemic risk effectively. This requires evaluating diverse outcomes and navigating contested priorities in a way that supports deliberation among affected actors. These capabilities extend beyond Tier 1 methods and require Tier 2 and Tier 3 approaches that incorporate multi‐criteria and participatory techniques.

Trade‐offs often define the core challenge of managing risk in pluralistic societies. For example, a drought mitigation strategy that reduces utility costs may increase other burdens on low‐income households (Rachunok and Fletcher 2023), while managed retreat may reduce exposure but disrupt social cohesion. Without frameworks to evaluate such tensions explicitly, assessments may produce technically sound but socially contested outcomes.

Methods for navigating these trade‐offs are emerging. Spatial multi‐criteria models demonstrate how different weightings produce distinct outcomes (Archie et al. 2025; Caparros‐Midwood et al. 2017). Metric choice research shows how weighting by asset value can favor affluent areas, whereas residence‐equivalent losses yield more equitable outcomes (D. R. Johnson and Geldner 2023; Markhvida et al. 2020). The spatial dimension complicates preference elicitation, as values vary across places, jurisdictions, and populations (Argyris et al. 2019; Keller and Simon 2019) requiring methods that are both analytically sound and procedurally fair (Ferretti and Montibeller 2019). Participatory mapping and co‐design processes from natural resource governance offer promising examples (Kyem 2021; Levine and Feinholz 2015; Montoya et al. 2021).

Ultimately, systemic risk assessment requires tighter integration with risk governance. Risk is co‐produced through engagement with multiple knowledge systems and value sets (Cremen et al. 2023; Kahan et al. 2012; Lofstedt 2012; Renn and Schweizer 2009). Frameworks emphasizing deliberation, iterative modeling, and shared learning (Bouder et al. 2013; Renn and Schweizer 2009; Schweizer 2021; Wang et al. 2026), alongside examples like Louisiana's Coastal Master Plan (D. R. Johnson 2021; Ramaswami et al. 2018), demonstrate how formal structures can embed diverse values into decisions. Co‐evolution of assessment methods and governance structures is essential for addressing systemic risk in ways that are both technically sound and socially legitimate (Rydén Sonesson et al. 2021).

### 3.3 From Correlation to Causal Reasoning for Intervention

Assessing systemic risk to inform risk reduction requires answering intervention questions: “What will happen if we act?” rather than observational questions: “What has happened?” This shift from describing patterns to understanding causal mechanisms determines whether interventions succeed or fail. Methods must explain how consequences propagate through systems and how actions alter those pathways.

This priority addresses the questions “What can go wrong?” and “How might our actions change the system?” In complex systems, causality is often opaque, shaped by non‐linear feedback loops and time lags. Tier 2 methods can trace branching cause–effect chains, but Tier 3 methods are needed to capture recursive dynamics and evolving system structure. These capacities are essential for evaluating intervention effects and trade‐offs under uncertainty.

Many assessment methods identify patterns: Areas that flooded repeatedly, populations disproportionately affected, infrastructure that failed during past events. These patterns reveal associations but not causes. They can show that low‐income communities experience greater losses, but not why. Losses could stem from building quality, emergency response capacity, insurance access, or other factors. Without understanding causal mechanisms, interventions may target factors that merely correlate with outcomes rather than cause them, wasting resources or even producing harm (S. E. Anderson et al. 2018; Lawrence, Blackett, et al. 2020; Pelling et al. 2022). System dynamics research has long shown that seemingly sensible solutions can backfire when applied without understanding how the systems work (Forrester 1971a; Rittel and Webber 1973). Without methods that reflect causal relationships, assessments may misrepresent not just what might happen, but how and why. As Haimes (2017) observed, risk analyses in complex systems often show what not to do, but fail to guide what should be done.

Tier 2 methods can trace structured chains of consequence, but Tier 3 methods are needed to capture feedback and emergence. Because controlled experiments are rarely possible, analysts rely on observational data and structured reasoning. Scenario simulations, causal inference techniques, counterfactual analysis and expert judgement can help map how interventions might propagate through systems (Allison et al. 2024; Cremen et al. 2022a; Lin et al. 2020).

The aim is to understand not just whether an intervention might work, but why and how, especially when effects vary across locations, unfold over time, or interact with other policies (D. R. Johnson and Geldner 2023). Machine learning can identify patterns but may not reveal the underlying processes needed for defensible intervention design (Guikema 2020; Hegde and Rokseth 2020). Conversely, qualitative tools like causal loop diagrams can visualize system structure but may lack the rigor needed to test specific interventions.

Approaches that combine participatory mapping with network or system dynamics modelling may enable more robust causal reasoning (Cradock‐Henry et al. 2020; Simpson et al. 2021; Cremen et al. 2022a; Allison et al. 2024; Hardaway and Flage 2025). Advancing these capabilities is essential for risk assessment to support intervention design, not simply describe potential consequences.

### 3.4 From Probability to Representing Uncertainty

Conventional risk assessment typically frames risk in terms of consequences and their probabilities. This formulation assumes uncertainties can be characterized probabilistically and that past patterns inform future likelihoods. In complex systems, this framing becomes inadequate. Here, risk must be understood as consequences and their associated uncertainties: Both measurable variability and knowledge limitations (Aven 2013b; Aven and Renn 2009)

This priority reframes the question “What is the likelihood?” for contexts where probability is insufficient. As assessments move from first‐order to higher‐order consequences, uncertainty deepens not just in magnitude but in nature. Methods must represent deeper forms of uncertainty: Incomplete knowledge, indeterminate futures, and unpredictable system behavior. Here, risk stems from interacting processes and evolving relationships, factors that are not just unknown but, in some cases, unknowable (Aven 2013b; Aven and Renn 2009; Flage et al. 2014; Marchau et al. 2019; Paté‐Cornell 1996).

A common distinction is made between aleatory uncertainty (inherent randomness) and epistemic uncertainty (incomplete knowledge) (Aven and Zio 2011; Flage and Aven 2015). Both pose challenges when uncertainty is deep. In such cases, assigning probabilities can obscure underlying ignorance rather than clarify it. Semi‐qualitative approaches (confidence levels, scenario spaces, structured expert judgment) may provide more transparent representations (Flage et al. 2014; Lempert et al. 2024).

The goal is not just to quantify uncertainty but to represent it in ways that inform decisions, support learning, and clarify what is known and what remains unresolved. Such representations can enhance transparency and usability, particularly in participatory or collaborative processes. Ideally, methods enable integration of these representations within adaptive frameworks, allowing assessments to remain valid as systems and knowledge evolve (Hardaway and Flage 2025; Raices Cruz et al. 2022).

Decision Making under Deep Uncertainty (DMDU) approaches, such as Dynamic Adaptive Policy Pathways (DAPP), Robust Decision Making, and Exploratory Modeling, move beyond reliance on precise predictions, instead supporting adaptive strategies that perform well across a range of plausible futures. These approaches emphasize flexibility and iterative learning, adjusting as systems evolve (Haasnoot et al. 2013/4; Lawrence, Haasnoot, et al. 2020; Lempert 2003; Marchau et al. 2019). While early applications relied on simplified models, advances now allow DMDU to be used with increasingly realistic simulations. Their goal is not precision but guidance under uncertain and changing conditions.

In some cases, the greatest challenge is not uncertainty within a known structure but transformation of that structure itself. Sudden shocks (pandemics, geopolitical conflict, disruptive technologies) can rapidly alter system behavior or introduce new threats (Xu et al. 2020). These shifts may render previous assessments obsolete, not because they were flawed, but because the system changed fundamentally. Addressing such transformation requires methods that are both adaptive and exploratory, capable of reasoning across discontinuous change and representing ignorance as well as knowledge. Integrating probabilistic techniques with approaches from foresight analysis and futures studies, such as scenario discovery, pathways analysis, and robust decision frameworks (Goodspeed 2020; Lempert 2003) can help assess not only what is likely, but what is plausible, or transformative. These approaches support decisions that remain valid even as systems evolve unexpectedly.

Ultimately, representing uncertainty is not a weakness of assessment but a reflection of system complexity and incompleteness of knowledge. Rather than masking uncertainty with false precision, effective methods must make it visible. This transparency lays the foundation for defensible assessments.

### 3.5 From Validity to Defensibility When Assessing Risk in Complex Systems

In complex, interdependent systems, traditional standards of model validation often no longer apply. Unlike simple physical systems, systemic risks unfold across interacting domains, evolve over time, and are shaped by uncertainty and value judgments. As the scope of assessment expands from direct risks to cascading and emergent ones, particularly in Tier 3 assessments, a shift is required: From seeking external validation to establishing defensibility. This means clarifying assumptions, representing diverse perspectives, and ensuring that assessments remain useful even when outcomes cannot be verified.

This priority addresses the question “What assumptions shape our model of the system?”, emphasizing that transparency, positionality, and framing choices become central to credibility. Analytical rigor remains important, but in complex systems, rigor does not equate to precision or perfect historical match. Because complex systems are stochastic, observed history represents one realization among many plausible pathways. A model that reproduces past events exactly may simply be overfit rather than capturing underlying dynamics. Data may be incomplete, outdated, or inconsistent across domains. Assessments often rely on assumptions that are rarely testable and may omit subtle but consequential interactions. Even well‐calibrated models of single components (e.g., bridge safety) may fail to capture the broader role of that component in maintaining system function (e.g., emergency access or supply chains). In such cases, defensibility means being transparent about what an assessment captures, and what it does not, and the underlying assumptions, acknowledging that outputs are contingent on framing, scope, and interdependencies. Rather than aiming for prediction or validation, assessments should aim to support structured exploration, clarify plausible scenarios, and surface trade‐offs for decision‐makers (Bristow and Mohareb 2020).

Good system risk assessments should be not only analytically defensible, but also socially and ethically defensible. This addresses the question of “Importance for whom?” Assessment approaches are not neutral tools but encode judgements about what matters, which risks count, and whose perspectives are represented. Every assessment reflects choices about scope, data sources, stakeholder inclusion, and what success looks like. These choices carry ethical weight, especially where risk is unevenly distributed and contested. Addressing this requires attention to procedural, recognitional, and distributional justice: Who shapes the model, whose values are embedded, and who bears the consequences (Soden et al. 2023). Transparent documentation, inclusive model development, and deliberate structuring of contestable assumptions are essential. Without this, technically sound assessments may lack social legitimacy or fail to support fair decisions (Argyris et al. 2019; McComas et al. 2016; Renn and Schweizer 2009; Williams et al. 2022).

Over time, all models degrade. Adaptive approaches, those that support revision, feedback, and stakeholder input maintain defensibility in dynamic conditions (Allison et al. 2024). But criteria for “good enough” remain unclear, especially under resource constraints. More importantly, who defines “good enough” raises ethical and political questions about who makes the determination and for whom.

Defensibility in complex systems is ultimately about transparency and humility, making clear what is known, what is assumed, and how things might change (Hardaway and Flage 2025). This ensures that risk assessments remain credible and useful, even amid complexity, uncertainty, and contestation.

### 3.6 Making Complexity Usable and Interpretable

Even the most advanced assessment methods offer limited value if their results cannot be understood or acted upon. As methods and models grow more sophisticated, capturing cascading effects and uncertainty, the challenge shifts from analysis to interpretation. Risk assessments increasingly fail not because models are wrong but because outputs are inaccessible, misused, or mistrusted. This final research priority focuses on how complexity is represented and communicated.

Tier 2 and Tier 3 assessments must convey interdependencies, uncertainty, and trade‐offs without oversimplifying. Too often, rich assessments are collapsed into simplified tools like risk matrices and heatmaps. These formats persist because they are intuitive, not because they are accurate. Such tools often distort relationships, conceal uncertainty, and misguide prioritization (Cox 2008).

The challenge is one of representation. Risk assessments must convey nuance without overwhelming audiences, especially when consequences are multidimensional and interpretations are contested (Bruine de Bruin et al. 2024; Peters et al. 2025; Stone et al. 2017). Communicating uncertainty is particularly difficult. Institutional pressures often favor clarity, even at the cost of transparency. Yet in complex systems, suppressing uncertainty can foster false confidence and poor decisions (Schneider et al. 2022; van der Bles et al. 2020). Assessment methods must be coupled with communication approaches that address this tension: How to present complexity in forms that decision‐makers can use without losing essential detail.

Meeting this challenge requires integrating risk assessment and risk communication as a two‐way process from the start (Árvai 2014; Balog‐Way et al. 2020; Stewart 2024), working with decision‐makers and stakeholders throughout, and developing tools that represent complexity and uncertainty without overwhelming recipients. Approaches such as layered visualizations, spatial uncertainty overlays, and participatory processes can connect formal analysis with local values. Moving from static reports to interactive platforms enables stakeholders to explore underlying data, update assumptions, and test scenarios, supporting iterative learning rather than one‐way information transfer. All such approaches must be grounded in risk communications practice and developed in collaboration with risk communications researchers.

Making assessments usable and interpretable is fundamental to their value. This requires treating communication as integral to design, collaborating with communication researchers from the start, and developing representations that decision‐makers can understand and trust. When assessments are accessible, they can inform policy, support community understanding, and facilitate dialogue in contested contexts (Filippi et al. 2023; Gentile et al. 2025; Reilly et al. 2018).

## 4 Future Directions

This paper identifies research priorities for developing methods to assess risk in complex, interdependent systems. We call for a shift in how risk methods are framed, evaluated, and integrated into decision processes. To support this shift, we introduced a taxonomy of Risk Assessment Tiers, classifying methods by their capability to assess different types of consequences and associated uncertainties. This framing clarifies when existing approaches are appropriate, where their limits lie, and how future techniques must evolve to address cascading and emergent risks. It provides a common structure for aligning method development, enabling researchers to build on one another's work.

We identify six priorities that follow from this foundation:

- Analyzing consequences: Develop methods that trace consequences across systems and orders of risk, capturing multidimensional impacts and causal complexity.
- Structuring uncertainty: Advance frameworks that represent epistemic and aleatory uncertainty (including deep uncertainty) across space, time, and model structure.
- Evaluating trade‐offs: Enable participatory, multi‐criteria assessments that reflect diverse values across spatial and temporal scales.
- Strengthening causal inference: Move from diagnostics to methods that support reasoning about interventions, feedbacks, and dynamic responses.
- Ensuring defensibility: Clarify model assumptions and framing, enable transparent debate, and support continuous revision.
- Representing results: Co‐design outputs with decision‐makers and communities using approaches grounded in risk perception and communication theory.

Our aim is to structure a coherent body of knowledge for systemic risk assessment, providing a foundation that future researchers and practitioners can build on and extend.

Advancing this agenda requires more than only improving models. It requires rethinking the role of the analyst, not as a detached evaluator, but as a systems interpreter and collaborator. Analysts must understand not only technical methods but the broader process of risk, how it is perceived, communicated, governed, and used. This means working across disciplinary and institutional boundaries to ensure risk information is interpreted with appropriate care. The goal is not to calculate risk with perfect precision, but to develop assessment methods that are robust, transparent, and adaptable: Capable of informing understanding and governance in a world of compounding, systemic risk.

## Author Contributions

**T. Logan**: conceptualization, formal analysis, investigation, methodology, project administration, visualization, writing – original draft, writing – review and editing, project administration. **B. Rachunok**: conceptualization, formal analysis, investigation, methodology, project administration, visualization, writing – original draft, writing – review and editing. **K. Hardaway**: conceptualization, formal analysis, investigation, methodology, project administration, visualization, writing – original draft, writing – review and editing. **D. Bristow**: conceptualization, formal analysis, investigation, methodology, project administration, visualization, writing – original draft, writing – review and editing. **A. Reilly**: conceptualization, formal analysis, investigation, methodology, project administration, visualization, writing – original draft, writing – review and editing. **D. Johnson**: conceptualization, formal analysis, investigation, methodology, project administration, visualization, writing – original draft, writing – review and editing. **J. Johansson**: conceptualization, formal analysis, investigation, methodology, project administration, visualization, writing – original draft, writing – review and editing. **G. Cremen**: conceptualization, formal analysis, investigation, methodology, project administration, visualization, writing – original draft, writing – review and editing. **J. Boakye**: conceptualization, formal analysis, investigation, methodology, project administration, visualization, writing – original draft, writing – review and editing. **P. Schweizer**: conceptualization, formal analysis, investigation, methodology, project administration, visualization, writing – original draft, writing – review and editing.

## Funding

The study was funded by HORIZON EUROPE Climate, Energy and Mobility (grant no. 101147385), Royal Society Te Apārangi: Rutherford Discovery Fellowship, and Ministry for Business Innovation and Employment.

## Conflicts of Interest

The authors declare no conflicts of interest.
